# High-resolution adaptive optics findings in talc retinopathy

**DOI:** 10.1186/s40942-015-0009-4

**Published:** 2015-07-24

**Authors:** Mohamed K Soliman, Salman Sarwar, Mostafa Hanout, Mohammad A Sadiq, Aniruddha Agarwal, Vikas Gulati, Quan Dong Nguyen, Yasir J Sepah

**Affiliations:** 1 Ocular Imaging Research and Reading Center, Stanley M. Truhlsen Eye Institute, University of Nebraska Medical Center, 985540 Nebraska Medical Center, Omaha, NE 68198-5540 USA; 2 Stanley M. Truhlsen Eye Institute, University of Nebraska Medical Center, Omaha, NE USA; 3 Department of Ophthalmology, Assiut University Hospital, Assiut University, Assiut, Egypt

**Keywords:** Adaptive optics, Talc retinopathy, Crystalline retinopathy

## Abstract

Talc retinopathy is a recognized ocular condition characterized by the presence of small, yellow, glistening crystals found inside small retinal vessels and within different retinal layers. These crystals can be associated with retinal vascular occlusion and ischemia. Different diagnostic modalities have been used previously to characterize the retinal lesions in talc retinopathy. Adaptive optics, a high resolution imaging technique, is used to evaluate the location, appearance and distribution of talc crystals in a case of talc retinopathy.

## Background

Talc retinopathy is a recognized ocular condition characterized by the presence of small, yellow, glistening crystals found inside small retinal vessels and within different retinal layers [1]. The crystals are thought to be secondary to emboli derived from talc which is an insoluble inert particulate filler material (excipient) used in preparation of certain oral (methylphenidate hydrochloride, methadone, pentazocine and amphetamine), inhalational (crack cocaine), and intravenous (cocaine and heroin) drugs [2, 3]. When these oral tablets are crushed and injected intravenously, most of the talc particles get trapped in the pulmonary vasculature except for very small particles (<7 μm) that escape from pulmonary capillary bed and reach the eye through systemic circulation [3, 4]. Ocular findings usually develop after chronic intravenous drug abuse and range from asymptomatic crystalline retinopathy to ischemic manifestations of capillary non perfusion and neovascularization [5]. Adaptive optics (AO) retinal imaging has been used to study retinal vessels in various retinal vascular diseases such as diabetic retinopathy and age-related macular degeneration. In this report, we used AO camera (rtx1; Imagine Eyes) and spectral domain optical coherence tomography to evaluate the location, appearance and distribution of talc crystals in a case of talc retinopathy.

## Case presentation

**Fig. 1:**
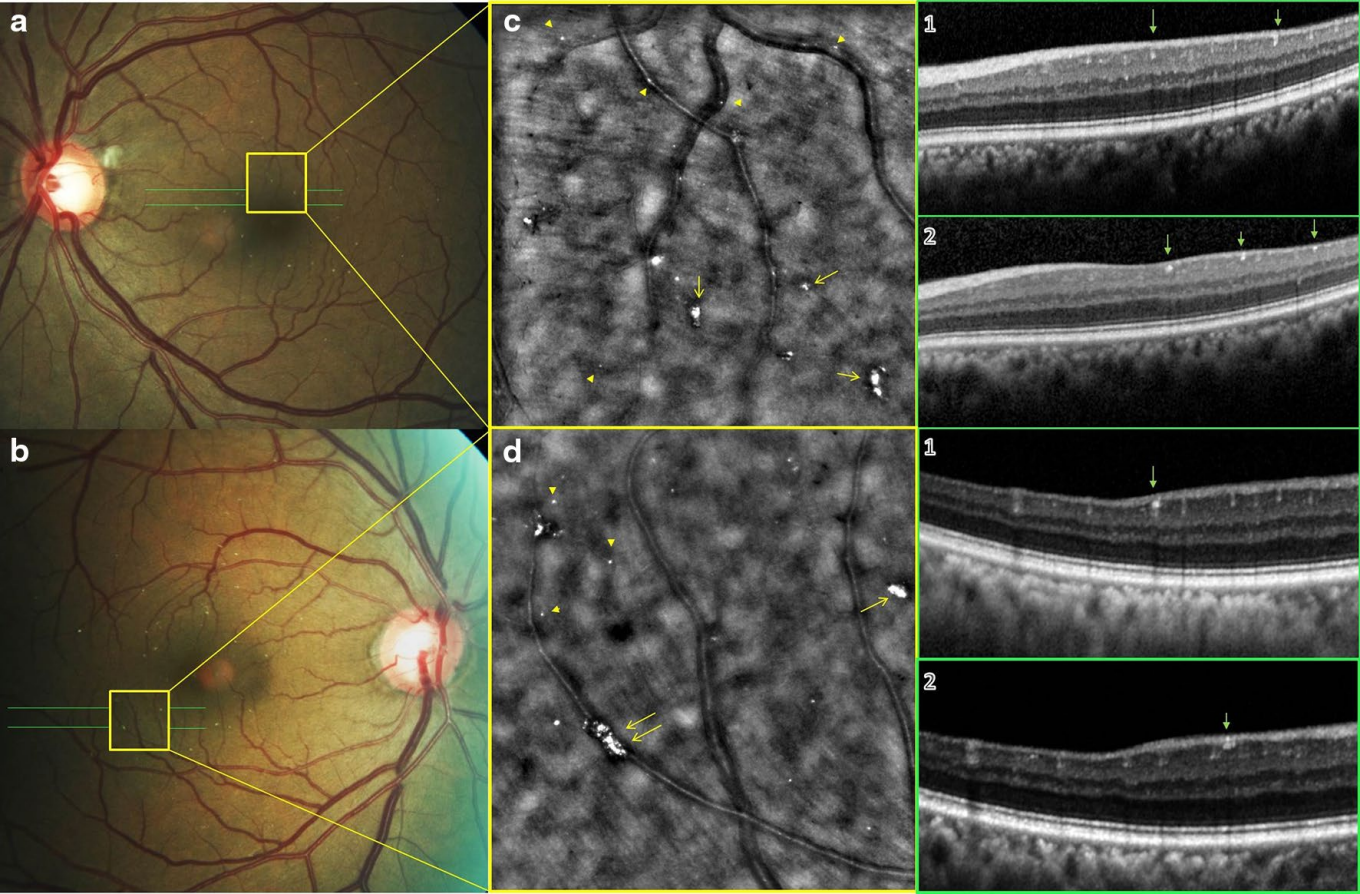
**a**, **b** Color fundus photographs of left and right eye showing multiple glistening small *yellow crystals* scattered intravascularly and extravascularly within the posterior pole. **c**, **d** Adaptive optics images of the two *yellow squares* marked in the color fundus photographs in **a**, **b**. *Arrows* point the clumps of talc particles seen in the color fundus photographs. *Arrow heads* point the tiny talc particles that could not be detected clinically. (*1*), (*2*) OCT scans corresponding to the 2 *green lines* in the color fundus photographs demonstrating the location of these talc crystals (hyper-reflective dots).

**Fig. 2:**
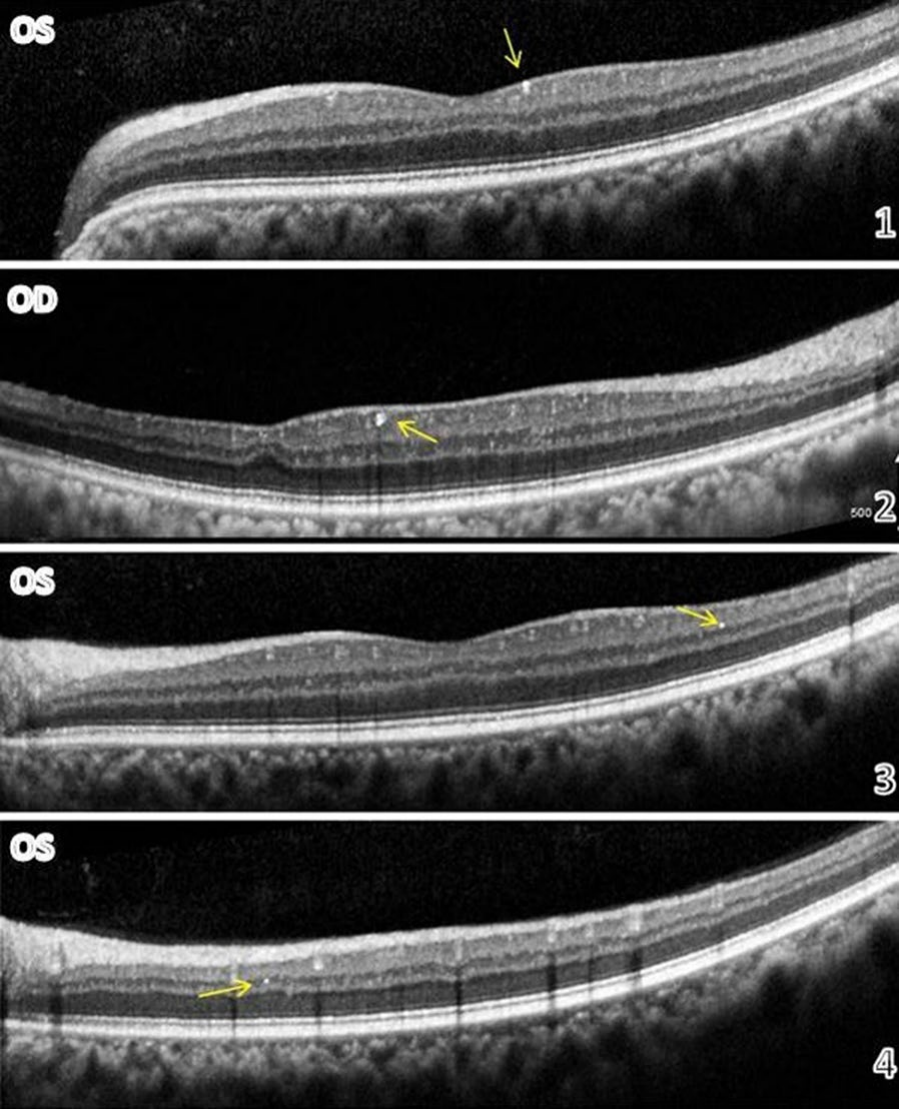
OCT scans through different areas of the retina showing the location of talc crystals in different retinal layers: Nerve fiber layer (*1*), Ganglion cell layer (*2*), inner plexiform layer (*3*) and inner nuclear layer (*4*). The second OCT scan (*2*) demonstrates thinning of the inner retinal layers.

A 55-year-old African American female with a past medical history of intravenous drug abuse (cocaine), chronic hepatitis B & C, chronic obstructive pulmonary disease (COPD) and hypertension presented with blurring of vision, and floaters. Best-corrected visual acuity was 20/25 and slitlamp biomicroscopy was unremarkable in both eyes. Intraocular pressure was 30 in the right eye and 28 in the left (Goldmann applanation tonometry). Fundus examination showed multiple, small, yellow, glistening crystals scattered throughout the posterior pole of both eyes. Crystals appeared to be located inside small retinal arterioles and venules of the macula with few crystals scattered outside the vessels within the retina (Fig. 1). The optic nerve showed increased cup to disc ratio of 0.75 with no disc hemorrhage OU. There were no signs of neovascularization and retinal periphery was unremarkable in both eyes. SD-OCT demonstrated multiple hyper-reflective dots of varying sizes scattered among the nerve fiber layer, ganglion cell layer, inner plexiform and inner nuclear layer (Fig. 2). Thinning of the inner retinal layers in temporal part of the macula, a finding similar to that usually seen with vascular occlusion was also noted in the right eye (Fig. 2). AO imaging revealed multiple shiny refractile dots distributed both intravascularly and extravascularly corresponding to those seen in color photo. The high resolution of AO allowed detection of some tiny particles that were not detectable clinically (Arrow heads: Fig. 1).

Different diagnostic modalities have been used previously to characterize the retinal lesions in talc retinopathy. Fundus examination usually reveals a characteristic appearance of these crystals, which are found inside the small retinal vessels and throughout the fundus. Talc crystals should be differentiated from other conditions causing crystalline retinopathy and from other causes of retinal embolism. Classification of the detected materials was based on clinical diagnosis. Careful examination of these crystals have shown that they most commonly represent an accumulation or clumps of multiple talc particles rather than a single embolus of talc [6]. Absence of retinal granuloma as may be seen in the lungs of IV drug users might be due to the blood retinal barrier. A vascular filling defect is seen on fluorescein angiography when the embolus block the small retinal blood vessels causing capillary non perfusion and retinal ischemia [5]. With the introduction of SD-OCT, the locations of these crystals with respect to retinal layers have been demonstrated. They were found distributed among the inner retinal layers where retinal blood vessels reside [1].

## Conclusion

In this report, SD-OCT has highlighted the structural damage of the retina as demonstrated by thinning of inner retinal layers most probably due to vascular occlusion as well as revealed the location of these crystals which were seen in all layers of the inner retina. AO allowed better elucidation of the clumps of the particles that form the talc microembolus with crystals clearly seen impacted inside the retinal arterioles and venules of the posterior pole as well as within the surface of the retina (Fig. 1) [6]. In addition, AO imaging enabled establishment of the extent of tissue involvement through detecting numerous tiny talc particles that were neither evident on clinical examination nor by OCT. Talc particles smaller than 5 μm in size are generally not discerned clinically and are beyond the resolution limits of conventional OCT. However, they were made visible using the high resolution AO imaging technique. Detection of these tiny particles revealed retinal involvement in this case to be far more severe than apparent clinically. Therefore, AO imaging can be used as a valuable tool for screening of current or past drug abuse prior to the development of retinopathy and appearance of talc crystals. Moreover, this finding may add new insights into the extent of retinal involvement in different retinal diseases, which may be more extensive than what we think it really is. This may lead to modification of diseases classifications and might contribute to changing treatment paradigm with earlier intervention.

### Consent

Written informed consent was obtained from the patient.

## Abbreviations

AO: adaptive optics
SD-OCT: spectral domain optical coherence tomography
